# Associations of genetic variation in *CASP*3 gene with noise-induced hearing loss in a Chinese population: a case–control study

**DOI:** 10.1186/s12940-017-0280-y

**Published:** 2017-07-24

**Authors:** Yinyin Wu, Juntao Ni, Mingjian Qi, Chengjian Cao, Yuxian Shao, Liangwen Xu, Haiyan Ma, Lei Yang

**Affiliations:** 1 Department of Preventive Medicine, School of Medicine, Hangzhou Normal University, Hangzhou 310018 Zhejiang, People’s Republic of China; 2 Hangzhou hospital for prevention and treatment of occupational diseases, Hangzhou 310014 Zhejiang, People’s Republic of China

**Keywords:** Genetic susceptibility, *CASP3*, Single-nucleotide polymorphism/SNP, Noise-induced hearing loss/NIHL

## Abstract

**Background:**

Noise-induced hearing loss (NIHL) is a complex disease caused by environmental and genetic risk factors. This study explored the relationship between the genetic variations in the *CASP* gene and the risk of developing NIHL among Chinese workers exposed to occupational noise.

**Methods:**

A case–control study of 272 NIHL workers and 272 normal-hearing workers matched for age, sex and years of noise exposure was conducted. Fifteen single-nucleotide polymorphisms (SNP) in the *CASP1*, *CASP3*, *CASP4, CASP5*, *CASP6*, *CASP8*, *CASP9*, *CASP10* and *CASP14* genes were genotyped using the polymerase chain reaction–ligase detection reaction method. Using conditional logistic regression models, the adjusted odds ratios (ORs) and 95% confidence intervals (CIs) of genetic variations associated with NIHL risk were calculated.

**Results:**

Two SNPs in the *CASP3* gene were associated with NIHL risk. For rs1049216, TT genotype was associated with a decreased risk of NIHL (OR = 0.246, 95% CI = 0.069–0.886) when compared with the CC genotype. For rs6948, the AC and CC genotype were associated with a decreased NIHL risk (OR = 0.568, 95% CI = 0.352–0.916) compared with AA genotype. There were joint effects of working time and *CASP3* polymorphisms on NIHL risk (*P* < 0.05).

**Conclusions:**

Genetic variations in the *CASP3* gene and the joint effects of working time and *CASP3* polymorphisms may modify the risk of developing NIHL.

**Electronic supplementary material:**

The online version of this article (doi:10.1186/s12940-017-0280-y) contains supplementary material, which is available to authorized users.

## Background

Long-term and high-intensity noise directly affects hearing and leads to noise-induced hearing loss (NIHL) [1]. Hearing loss is the most prevalent sensory organ disability worldwide. The World Health Organization (WHO) has estimated that the number of people with hearing disability was 42,000 in 1985, 120 million in 1995,250 million in 2001, and up to 360 million in 2011 [2]. In the United States, 10 million people have NIHL, and in China, the number of workers with NIHL has increased by 77.8% from 2010 to 2012 [3].

Besides noise exposure, there are many other risk factors of NIHL such as smoking, alcohol consumption, organic solvent exposure, high blood pressure, cholesterol, and sleeping problems [4, 5]. Genetic factors and gene-environment interactions might also play an important role in the development of NIHL. Researchers have observed that individuals have different outcomes of hearing damage, even exposed to similar levels of noise [6, 7].

The occurrence of NIHL is based on cochlear hair cell damage and death [8]. Apoptosis is one of the ways to cause hair cell death, which is a cell-independent and orderly death controlled by a specific gene [9, 10]. Caspase activation is the central link in apoptosis. Caspases are activated by stepwise hydrolysis under the influence of an apoptotic signal, and cleavage of cell structural and functional proteins causes apoptosis [11]. Many species of caspase have been founded, and can be divided into three categories, according to difference of cascade reaction in the upstream and downstream position and function. The first class is the apoptotic promoters, including caspase-8, −9 and −10, which are located upstream of the cascade reaction, and can self-activate and activate downstream caspases. The second class is the apoptotic effectors, including caspase-3 and -6, which are located downstream of the cascade reaction, and can be activated by the upstream promoter. Caspases that have been activated can act on the specific substrate to cause biochemical and morphological changes in cells, leading to apoptosis. The third class is associated with inflammation and plays a supporting role in death-receptor-mediated apoptosis, and includes caspase-1, −4, −5 and −14.

Given the important roles of caspases in apoptosis, we assumed that variants of these genes might be associated with the risk of NIHL in the Chinese population. To examine this hypothesis, we genotyped 15 single-nucleotide polymorphisms (SNP) in the *CASP1*, *CASP3*, *CASP4*, *CASP5*, *CASP6*, *CASP8*, *CASP9*, *CASP10* and *CASP14* genes in 272 NIHL workers and 272 normal-hearing workers, and analyzed the associations of these SNPs with NIHL. We also explored the interactions among these SNPs and noise exposure.

## Methods

### Participants

This study involved 272 NIHL workers and 272 normal-hearing workers who were recruited from a cross-sectional study of 1549 occupational noise-exposed workers, which was conducted between March 1 and December 31, 2014. These workers were exposed to continuous and steady occupational noise in 16 factories, such as machinery manufacturing and a thermal power plant of Hangzhou City, Zhejiang Province. None of the workers was exposed to other occupational hazards. The inclusion criteria for participants were as follows: (1) cumulative time of occupational noise exposure [noise exposure time ≥ 8 h/day or 40 h/week, and noise intensity ≥80 dB(A)] >1 year, and (2) Han ethnicity. Participants with a history of exposure to explosives, head injury, family history of hearing loss, other otological diseases, otitis, fever or common infections (e.g., influenza, diarrhea and hepatitis), or with ototoxic drug administration within 1 month before the physical examination were all excluded.

Participants were selected and divided into cases and controls according to the following criteria. The definitions of the cases were: (1) participants with normal hearing before exposure; (2) >1 year of occupational noise exposure; and (3) hearing threshold worse than 25 dB at high frequency. The definitions of the controls were: (1) participants >1 year of occupational noise exposure; and (2) hearing thresholds < 25 dB at each frequency. The controls were matched with sex, age (±5 years), and years of noise exposure one for each case.

The study was approved by the Research Ethics Committees of Hangzhou Institute for Occupational Health with informed consent obtained from each participant.

### Physical examination and epidemiological investigation

Trained physicians performed a physical examination following a standard protocol for each participant, and parameters including body weight and height, pulse rate, and blood pressure were collected. Trained professional physicians conducted a face-to-face interview by using a structured questionnaire to collect information from each participant, including demographic characteristics, lifestyle habits, history of disease and drug use, family history of deafness, history of noise exposure in the workplace, and use of ear protection for noise.

### Audiological status assessment and environmental noise measurement

Before audiometry, all participants stopped noise exposure for >48 h. Audiometry was carried out by trained physicians using standard procedures for each participant in a sound-attenuating chamber with a background noise level < 25 dB(A). According to the Chinese Diagnostic Criteria of Occupational NIHL (GBZ49–2014), hearing thresholds of both ears were determined with the ascending method in 5-dB steps at frequencies of 500, 1000, 2000, 3000, 4000 and 6000 Hz. The results were polished for age and gender according to GB/T7582–2004. The hearing threshold at high frequency by PTA was defined as the average at 3000, 4000 and 6000 Hz for each ear. The hearing threshold at speech frequency was defined as the average at 500, 1000 and 2000 Hz for each ear.

A noise statistical analyzer (AWA6218; Westernization Instrument Technology Co. Ltd., Beijing, China) was used to evaluate the intensity of noise in the workplace. Noise exposure was evaluated with continuous dB(A)-weighted sound pressure levels (Lex.8 h) according to the National Criteria of Measurement of Noise in the Workplace (GBZ/T189.8–2007) (China, 2007). Cumulative noise exposure (CNE) was calculated as CNE = Lex.8 h + 10log^T^, where T means years of noise exposure.

### SNP selection and genotyping

Candidate SNPs in the *CASP1*, *CASP3*, *CASP4*, *CASP5*, *CASP6*, *CASP8*, *CASP9*, *CASP10* and *CASP14* genes were selected based on HapMap database (http://hapmap.ncbi.nlm.nih.gov), dBSNP (http://www.ncbi.nlm.nih.gov/snp/), and 1000 genomes dataset (http://www.1000genomes.org/). SNP function prediction software was also adopted such as Variant Effect Predictor (http://asia.ensembl.org/info/docs/tools/vep/index.html) and SNP Function Prediction (http://snpinfo.niehs.nih.gov/snpinfo/snpfunc.htm). Inclusion criteria were as follows: (1) located in the coding region, promoter region, 3′-untranslated region (UTR), or 5′-UTR; (2) minor allele frequency (MAF) of Chinese Han living in Beijing (CHB) >0.10; (3) linkage disequilibrium value of r^2^ > 0.80; and (4) reported in previous studies [12–15]. Fifteen SNPs were selected according to those criteria: rs1977989 in *CASP1*, rs1049216 and rs6948 in *CASP3*, rs630003 in *CASP4*, rs507879 and rs523104 in *CASP5*, rs1042891 in *CASP6*, rs1045494 and rs3769823 in *CASP8*, rs1052576 and rs4645978 in *CASP9*, rs13006529 and rs3900115 in *CASP10*, and rs3181312 and rs3181309 in *CASP14*.

Genomic DNA was isolated from peripheral blood samples using TIANamp Blood DNA Kits (Tiangen Biotech, Beijing, China) and was stored at −80 °C. Genotyping of the SNPs was carried out using the polymerase chain reaction–ligase detection reactions (PCR-LDR) method (Shanghai Generay Biotech Company). The primer and probe sequences are shown in Table 1.

**Table 1:** Basic information, primer and probe sequences of selected SNPs

No.	Gene	rs No.	location	allele	MAF (CHB)	Primers for PCR	Probes for detecting variations	*P*_HWE_
1	*CASP1*	rs1977989	11:105,026,117	A/G	0.232	F:TGAATATCATGTGGGCGCTC R:TTTCTACCTCTTCCCAGGAC	TA:GGATTTTAGAGCATTTCAAAATTCA TG:TTTGGATTTTAGAGCATTTCAAAATTCG TR:AATTTTTGGATTAAGGATTCTCAGC	0.325
2	*CASP3*	rs1049216	4:184,628,935	C/T	0.2	F:GTGCAGTCATTATGAGAGGC R:TTTGAGCCTTTGACCATGCC	TC:TTTTTTTTTGAAAAAGTTAAACATTGAAGTAAC TT:TTTTTTTTTTTTGAAAAAGTTAAACATTGAAGTAAT TR:GAATTTTTATGATATTCCCCCCACTTTTTTT	0.279
3	*CASP3*	rs6948	4:184,627,976	A/C	0.16	F:TGTTCTAAAGGTGGTGAGGC R:TGAGACTTGGTGCAGTGACG	TA:TTTTTTTTTTTTTCCAGCCGGAGGCCAGAGCTGAGCA TC:TTTTTTTTTTTTTTTTCCAGCCGGAGGCCAGAGCTGAGCC TR:CTCAGCCCGGGAGGCAGGCTCCAGGTTTTTTTTT	0.264
4	*CASP4*	rs630003	11:104,970,269	G/C	0.25	F:CTTCTGCCTGGTTGATTTGG R:GCTGAGATGGATGAACTGAC	TC:TTTTGATACTTGTGTTTGAATCATGAAGC TG:TTTTTTTGATACTTGTGTTTGAATCATGAAGG TR:TCTCACGCTGTGTTTCTCAGCTCCATTT	0.911
5	*CASP5*	rs507879	11:105,007,200	A/G	0.329	F:GTTAAGATGTTGGAATACCTG R:GATCTTTTGGTCCATATTGAG	TA:TTTTTTTTGAGGAAAAGAAAAAATATTATGATA TG:TTTTTTTTTTTGAGGAAAAGAAAAAATATTATGATG TR:CCAAAATTGAAGACAAGGCCCTGATTTTTTT	0.522
6	*CASP5*	rs523104	11:104,998,981	G/C	0.211	F:GCCTATCTTGATTTCATAGAA R:AGAAAAACATGGGGAACTCTG	TC:TTTTTTTTTTTTTCTCAGATGACTGTGAAGAGATGAC TG:TTTTTTTTTTTTTTTTCTCAGATGACTGTGAAGAGATGAG TR:TGCCAAGGATGCTGGAGAGTCTCTGTTTTTTTTT	0.623
7	*CASP6*	rs1042891	4:109,688,930	C/T	0.483	F:AATCCCAGCTACTTGGAAGG R:TAAGGCAGTTCTCTGCTAGG	TC:TCGTTAGGGTGAAGCATTATGGTCC TT:TTTTCGTTAGGGTGAAGCATTATGGTCT TR:AATGATTCAAATGTTTTAAAGTTTA	0.581
8	*CASP8*	rs1045494	2:201,287,058	T/C	0.183	F:CTGGCCGATGGTACTATTTAG R:TAAGCCCTCTATATTCATAGC	TC:TTTTTTTTTTTTTTTTAATAACACTGTCTCCTTTCTCTTAC TT:TTTTTTTTTTTTTTTTTTTAATAACACTGTCTCCTTTCTCTTAT TR:GCTTAAGGCTTTGGGAATGTTTTTAGCTGGTTTTTTT	0.522
9	*CASP8*	rs3769823	2:201,258,272	C/T	0.366	F: ATATTGTGGTTTCCTGTTGAAG R: TAGTCAACTCAACAGGAAGTG	TC:AAGGCTCCCAGGAAGAAGTTTCTTC TT:TTTAAGGCTCCCAGGAAGAAGTTTCTTT TR:TACTTTCAATAACCACCCTGGCTCT	<0.001
10	*CASP9*	rs1052576	1:15,506,048	G/A	0.366	F:TGCTTCTGGCTCACCTGCAGC R:TTGGACACAAGAAGGGACATG	TA:TTTTTGCTGGCTTTGCTGGAGCTGGCGCA TG:TTTTTTTTGCTGGCTTTGCTGGAGCTGGCGCG TR:GCAGGACCACGGTGCTCTGGACTGCTTT	0.802
11	*CASP9*	rs4645978	1:15,525,539	A/G	0.392	F:GAAAAGTGGCTAGCACGATG R:CTGTTCCAAACTCGAGTGTC	TA:GTGTGGGAGGTGTTAGGGTGGGAGA TG:TTTGTGTGGGAGGTGTTAGGGTGGGAGG TR:GAGGGAAGAGGGAATGGAAGACTGT	0.324
12	*CASP10*	rs13006529	2:201,217,736	A/T	0.2	F:CTGCTGTCAACGATGATGTG R:CTAGAATGACAGGTGGAGAC	TA:CCTGTGCCCCTGGATGCACTTTCAA TT:TTTCCTGTGCCCCTGGATGCACTTTCAT TR:TATAGCAGAGAGTTTTTGTTGGTTC	0.452
13	*CASP10*	rs3900115	2:201,185,954	A/G	0.22	F:GCTGGCCATGAAATCTCAAG R:AAAGGGTCTTCCTCACTCAG	TA:TTTTGCTGGAGAAGTCCAGCTCAGCCTCA TG:TTTTTTTGCTGGAGAAGTCCAGCTCAGCCTCG TR:GATGTTTTTGAACATCTCTTGGCAGTTT	0.341
14	*CASP14*	rs3181312	19:15,057,642	A/C	0.466	F:CCTCAGCTTCTCATATCTGC R:TTAACTCCTGGCCTCAAGTG	TA:TTTTAGTGGCTCACACCTGTATTCCCGAA TC:TTTTTTTAGTGGCTCACACCTGTATTCCCGAC TR:TTTGAGAGTCTGAAGCGGGAGGATCTTT	0.272
15	*CASP14*	rs3181309	19:15,056,984	T/C	0.279	F:ATTCCCTGTTGTCACCTTGC R:AGCTCCTACCTTGGAGATAG	TC:CTCTTTTCCACAACCTGCTAAATCC TT:TTTCTCTTTTCCACAACCTGCTAAATCT TR:TGATCCATCTGAAAACTTTTCTAAT	0.136

The PCR was performed in an ABI Prism 7000 Sequence Detection System in a total volume of 15 μl, including 1 μl genomic DNA, 1.5 μl 10× PCR buffer, 1.5 μl MgCl_2_, 0.3 μl dNTPs, 0.15 μl each primer, and 0.2 μl Taq DNA polymerase. The PCR was performed as follows: an initial melting step of 3 min at 94 °C, 35 cycles of denaturation for 15 s at 94 °C, annealing for 15 s at 55 °C and extension for 30 s at 72 °C, followed by a 3 min final extension at 72 °C. The ligation reaction for each PCR product was carried out with a total volume of 10 μl, including 3 μl PCR product, 1 μl 10× Taq DNA ligase buffer, 5 U Taq DNA ligase, and 0.01 μl each discriminating probe. The LDR was performed as follows: 30 cycles at 94 °C for 30 s and 56 °C for 3 min. After the LDR reaction, 1 μl LDR product was mixed with 8 μl loading buffer, and was melted for 3 min at 95 °C. The mixture was then analyzed on the ABI3730xl platform. Ten percent of the samples were randomly selected and genotyped repeatedly for the quality control, and the concordance was 100%.

### Statistical analysis

Continuous variables in accordance with normal distribution were expressed as mean ± standard deviation (SD) and were analyzed by Student’s *t*-test. Continuous variables for skewed distribution were expressed as median (P25, P75) and were analyzed by Wilcoxon rank test. Categorical variables were expressed as frequencies (%) and were analyzed by Person’s χ^2^ test. Hardy–Weinberg equilibrium tests were conducted using Pearson’s χ^2^ test for each SNP among the controls. The associations between the SNPs and NIHL risk were evaluated using conditional logistic regression for adjusted odds ratio (OR) with 95% confidence interval (CI). Potential gene–environment interaction was explored by stratified and crossover analysis. Multifactor dimensionality reduction (MDR) analysis was conducted to explore gene-gene interaction using MDR version 1.0.0 (Computational Genetics Laboratory of the University of Pennsylvania, Philadelphia, PA, USA). All the statistical analyses were performed using SAS version 9.1 (SAS Institute, Cary, NC, USA). *P* < 0.05 was considered statistically significant. Bonferroni correction was applied for multiple hypothesis testing.

## Results

The basic characteristics of the participants are shown in Table 2. There were 272 cases and 272 controls with an average age of 32.5 and 31.7 years, respectively. There were no significant differences between the cases and controls in the distribution of demographic characteristics, smoking habit, weekly working time, years of noise exposure, noise intensity exposure, and CNE (*P* > 0.05). Significant differences were observed for several lifestyle factors including music listening time, telephone use time, and time to go to sleep between the cases and controls (*P* < 0.05). Compared with the controls, more cases listened to music for >2 h/day, used the telephone for >16 min/day, and went to sleep later than 23:00 h.

**Table 2:** Basic characteristics of the study participants

	Controls(*n* = 272)	Cases(*n* = 272)	*χ* ^*2*^	*P*
Age (years, mean ± SD)	31.70 ± 7.67	32.48 ± 7.58	−1.192^a^	0.234
Sex, n (%)			0	1
Male	241(88.6)	241(88.6)		
Female	31(11.4)	31(11.4)		
Marriage status, n (%)			0.427	0.808
Single	69(25.4)	64(23.5)		
Married	201(73.9)	205(75.4)		
Divorce	2(0.7)	3(1.1)		
Education, n (%)			1.890	0.389
Primary school and below	96(35.3)	110(40.4)		
Junior middle school	123(45.2)	118(43.4)		
High school and above	53(19.5)	44(16.2)		
Income [yuan/month, n (%)]			2.488	0.288
< 2500	104(38.2)	118(43.4)		
2500-	79(29.0)	81(29.8)		
≧3500	89(32.7)	73(26.8)		
Smoking, n (%)			1.843	0.175
No	187(68.8)	172(63.2)		
Yes	85(31.2)	100(36.8)		
Music listening time (h/day)			17.770	<0.001
0	174(64.0)	130(47.8)		
< 2	88(32.4)	115(42.3)		
≧2	10(3.7)	27(9.9)		
Telephone using time (min/day)			27.224	<0.0﻿01
0	51(18.8)	20(7.7)		
< 16	142(52.2)	120(44.1)		
≧16	79(29.0)	131(48.2)		
Time go to sleep			48.818	<0.0﻿01
Early than 22:00	128(47.1)	56(20.6)		
22:00~	77(28.3)	86(31.6)		
Later than 23:00	67(24.6)	130(47.8)		
Working time (h/week)^b^	48.00(40.00–60.00)	50.00(40.00–60.00)	−1.370^c^	0.171
Noise exposure time (years)^b^	5.13(3.02–8.96)	5.04(3.00–8.50)	−0.046^c^	0.963
Noise intensity exposure, dB(A)^b^	83.20(80.00–87.40)	83.65(80.00–87.40)	−0.744^c^	0.457
CNE^b^	90.86(85.48–95.49)	91.13(86.59–96.15)	−0.665^c^	0.506

^a^Independent *t*-test

^b^Data was presented as median (P25, P75)

^c^Wilcoxon rank test

As shown in Table 1, 14 SNPs were in Hardy–Weinberg equilibrium (*P* > 0.05), except for rs3769823 of *CASP8*. This SNP was therefore excluded from the following analysis (Table 3). Associations were only found between rs1049216 and rs6948 and NIHL risk. For rs1049216, TT genotype was associated with a decreased NIHL risk (OR = 0.246, 95% CI = 0.069–0.886) when compared with the CC genotype. Similar results were found for the dominant and recessive genetic models. For rs6948, AC/CC genotypes were associated with a decreased NIHL risk (OR = 0.568, 95% CI = 0.352–0.916) when compared with the AA genotype. We classified rs1049216 CC and rs6948 AA genotypes as the high-risk genotypes. By computing the numbers of the two genotypes within the combined genotypes, we found that those carrying two risk genotypes were at higher risk of NIHL when compared with those carrying no risk genotype (OR = 1.787, 95%CI = 1.104–2.892). However, none of the associations remained significant after Bonferroni correction.

**Table 3:** Association between SNPs and NIHL risk

SNP	Genetic model	Genotype	Controls, n (%)	Cases, n (%)	P	OR(95%CI)^a^
rs1049216	Additive	CC	173(64.3)	195(72.5)		1.0
		CT	82(30.5)	68(25.3)	0.050	0.609(0.372–1.000)
		TT	14(5.2)	6(2.2)	0.032	0.246(0.069–0.886)
	Dominant	CC	173(64.3)	195(72.5)		1.0
		CT + TT	96(35.7)	72(27.5)	0.014	0.551(0.343–0.885)
	Recessive	CC + CT	258(94.8)	266(97.8)		1.0
		TT	14(5.2)	6(2.2)	0.047	0.277(0.078–0.986)
rs6948	Additive	AA	177(65.8)	198(73.6)		1.0
		AC	79(29.4)	65(24.2)	0.050	0.607(0.369–1.000)
		CC	13(4.8)	6(2.2)	0.089	0.328(0.091–1.185)
	Dominant	AA	177(65.8)	198(73.6)		1.0
		AC + CC	92(34.2)	71(26.4)	0.020	0.568(0.352–0.916)
	Recessive	AA + AC	256(95.2)	263(97.8)		1.0
		CC	13(4.8)	6(2.2)	0.128	0.373(0.105–1.327)
Risk genotype^b^		0	91(33.8)	71(26.4)		1.0
		1	6(2.2)	3(1.1)	0.701	0.684(0.098–4.779)
		2	172(63.9)	195(72.5)	0.018	1.787(1.104–2.892)

^a^adjusted for age, sex, education, marriage status, income, working time, noise exposure time, noise intensity exposure, CNE, telephone using time, music listening time, and time go to sleep

^b^rs1049216 CC and rs6948 AA genotypes were classified as high-risk genotypes; the number represents the numbers of the two genotypes within the combined genotypes

We also conducted stratified analysis by noise intensity and CNE, and found that when noise intensity was <85 dB(A) or CNE <90 dB(A), rs1049216 CC and rs6948 AA genotypes could be defined as high risk. When noise intensity was <85 dB(A), CT/TT genotypes of rs1049216 was associated with a decreased NIHL risk (OR = 0.425, 95% CI = 0.207–0.871) when compared with the CC genotype, and AC/CC genotypes of rs6948 were associated with a decreased NIHL risk (OR = 0.458, 95% CI = 0.227–0.926) when compared with the AA genotype. The risk of NIHL in those carrying two risk genotypes was 2.307-fold higher than in those carrying no risk genotype (OR = 2.307, 95%CI = 1.123–4.740). Similar results were found when CNE was <90 dB(A). However, none of the results reach statistical significance after Bonferroni correction (see Additional file 1: Table S1 and S2).

To explore further the potential gene–environment interaction in NIHL risk, we conducted a crossover analysis between the SNPs and nongenetic factors including working time, noise exposure time, noise intensity exposure, CNE, telephone use time, music listening time, and time to go to sleep. Associations were found between rs1049216 and rs6948 and working time. Individuals carrying rs1049216CC genotype and working >48 h/week were associated with a significantly higher risk of NIHL (OR = 3.989, 95% CI = 1.787–8.907) when compared with those carrying rs1049216 CT/TT genotype and working ≤48 h/week (Table 4). We found similar results for those carrying the CC genotype and not working >48 h/week (OR = 1.870, 95% CI = 1.012–3.457). Similar results were found for rs6948 and working time, as well as risk genotype and working time. When an individual carried the rs6948 AA genotype or had two risk genotypes, working time > 48 h/week showed a significant association with NIHL risk. We also conducted MDR analysis to explore the interaction between SNPs, but found no positive results (see Additional file 1: Table S3).

**Table 4:** Crossover analysis of interaction between SNPs and working time on NIHL risk

SNP	Genotype	Working time(h/week)	Controls, n (%)	Cases, n (%)	P	OR(95%CI)^a^
rs1049216	CT + TT	≤ 48	59(21.9)	43(16.0)		1.0
	CC	≤ 48	95(35.3)	91(33.8)	0.046	1.870(1.012–3.457)
	CT + TT	> 48	37(13.8)	31(11.5)	0.083	2.263(0.900–5.689)
	CC	> 48	78(29.0)	104(38.7)	0.001^b^	3.989(1.787–8.907)
rs6948	AC + CC	≤ 48	56(20.8)	42(15.6)		1.0
	AA	≤ 48	98(36.4)	92(34.2)	0.087	1.716(0.925–3.184)
	AC + CC	> 48	36(13.4)	29(10.8)	0.123	2.076(0.821–5.251)
	AA	> 48	79(29.4)	106(39.4)	0.001^b^	3.817(1.710–8.522)
Risk genotype	< 2	≤ 48	60(22.3)	43(16.0)		1.0
	2	≤ 48	94(34.9)	91(33.8)	0.045	1.875(1.014–3.467)
	< 2	> 48	37(13.8)	31(11.5)	0.082	2.267(0.902–5.699)
	2	> 48	78(29.0)	104(38.7)	0.001^b^	3.995(1.790-8.919)

^a^adjusted for age, sex, education, marriage status, income, noise exposure time, noise intensity exposure, CNE, telephone using time, music listening time, and time to go to sleep

^b^the *P*-value remained significant after Bonferroni adjustment for multiple comparisons

## Discussion

We investigated the association between caspase polymorphisms and NIHL in Chinese of Han nationality. We found significant differences between NIHL cases and controls for genotypic distributions of SNPs rs1049216 and rs6948 in *CASP3*. In rs1049216, compared with the participants carrying CC genotypes, the carriers with TT genotype had a decreased risk of NIHL (OR = 0.246, 95% CI = 0.069–0.886). In rs6948, the AC and CC carriers had decreased NIHL risk (OR = 0.568, 95% CI = 0.352–0.916) compared with the participants with AA genotype.

Caspase-3 is the most important effector in the process of apoptosis, and it is the converging point of many apoptotic stimulating signals. Its activation is a sign of apoptosis entering an irreversible stage [16, 17]. Many studies have investigated caspase-3 polymorphism and the risk of some diseases, including mainly Kawasaki disease and cancer, but few studies have related to NIHL [18, 19].

In our study, we defined rs1049216 CC and rs6948 AA genotypes as the high-risk genotypes. By calculating the numbers of the high-risk genotype, we found that the risk of NIHL in those carrying two risk genotypes was 1.787-fold higher than in those carrying no risk genotype (OR = 1.787, 95%CI = 1.104–2.892). This is similar to the results of Yan et al. [20]. By the stratified analysis, positive results were only founded when noise intensity was <85 dB(A) or CNE < 90 dB(A), and the effects of high-risk genotype were not observed when noise intensity was ≥85 dB(A) or CNE ≥90 dB(A). This result suggests that both SNPs are NIHL susceptibility sites and high-intensity noise is still a major NIHL risk factor. To address the question of whether there is a potential interaction between gene and environment, we conducted a crossover analysis between the SNPs and nongenetic factors. Associations were found between rs1049216 and rs6948 and working time. The risk of NIHL in individuals carrying the rs1049216CC genotype and working >48 h/week was 3.989-fold higher than in those carrying rs1049216 CT/TT genotype and working ≤48 h/week (OR = 3.989, 95% CI = 1.787–8.907). Similar results were found for those carrying the CC genotype and not working >48 h/week (OR = 1.870, 95% CI = 1.012–3.457), Similar results were found for rs6948 and working time, as well as risk genotype and working time. When individuals carried rs6948 AA genotype or two high-risk genotypes, working time > 48 h/week showed a significant association with NIHL risk. The results suggested that individuals with rs1049216 and rs6948 mutant alleles in the general population should pay more attention to controlling working time in order to achieve individualized control.

A previous study has shown that there was a strong linkage disequilibrium (D’0.98) between rs6948 and rs1049216, which was in agreement with our results (D’0.993) [21]. SNP’s located in the 3’UTR region play an important role in regulating the stability of mRNAs, mediating mRNA localization, assisting in identifying special codons, and controlling the level of mRNA translation [22, 23]. Both rs6948 and rs1049216 are located in the 3′UTR [24]. It has been suggested that mutant C allele of rs6948 in the binding site of miRNA-24 (binding site: *CASP3* 3’UTR1290–1296) may downregulate the expression of miRNA-24 and affect expression of caspase-3 [25]. Similarly, rs1049216 may also influence the expression of *CASP3* by controlling mRNA translation levels.

Our study had some limitations that should be acknowledged when interpreting our results. First, although this study adopted 1:1 matched case–control design, the sample size was small, and some positive results that could only have been revealed by large samples may not have been found. Second, all of the workers who had been diagnosed with occupational NIHL had been transferred from the original workplace. This study failed to incorporate them, which may have led to selection bias. Finally, due to limited conditions, we use factory, fixed-point measurement of noise level for individual noise exposure values, which may have affected the accuracy of the results.

## Conclusion

In conclusion, our findings suggest that genetic variations in the *CASP3* gene and their interactions with working time may modify the risk of developing NIHL. The TT genotype of rs1049216 and the AC and CC genotype of rs6948 may be less susceptible to NIHL. In subsequent studies, we will refine the experimental design, and supplement the collection of ambient noise exposure in a larger population to verify the results of the present study.

## Additional file

Additional file 1: Table S1. Stratified analysis by noise intensity. **Table S2.** Stratified analysis by CNE. **Table S3.** The best combination models identified by MDR. (DOCX 21 kb)

## Abbreviations

CHB: Han Chinese individuals from Beijing
CI: Confidence interval
CNE: Cumulative noise exposure
MAF: Minor allele frequency
MDR: Multifactor dimensionality reduction
NIHL: Noise-induced hearing loss
OR: Odds ratio
PCR-LDR: Polymerase chain reaction–ligase detection reactions
PTA: Pure tone audiometry
SD: Standard deviation
SNP: Single-nucleotide polymorphism
UTR: Untranslated region
WHO: World Health Organization
