# Supplementary material for: Associations of genetic variation in CASP3 gene with noise-induced hearing loss in a Chinese population: a case–control study
Source: Environ Health. 2017 Jul 24;16:78. doi: 10.1186/s12940-017-0280-y (PMC5525200; doi:10.1186/s12940-017-0280-y)
Supplement: Additional file 1: Table S1. — Stratified analysis by noise intensity. Table S2. Stratified analysis by CNE. Table S3. The best combination models identified by MDR. (DOCX 21 kb) [file 12940_2017_280_MOESM1_ESM.docx]

Table S1 Stratified analysis by noise intensity

| SNP | Genotype | Controls, n (%) | Cases, n (%) | P | OR(95%CI)^a^ |
| --- | --- | --- | --- | --- | --- |
| Noise intensity <85 dB(A) | | | | | |
| rs1049216 | CC | 95(61.7) | 112(76.2) |  | 1.0 |
|  | CT+TT | 59(38.3) | 35(23.8) | 0.019 | 0.425(0.207–0.871) |
| rs6948 | AA | 97(63.0) | 114(77.6) |  | 1.0 |
|  | AC+CC | 57(37.0) | 33(22.4) | 0.030 | 0.458(0.227–0.926) |
| Risk genotype | 0 | 57(37.0) | 33(22.4) |  | 1.0 |
|  | 1 | 2(1.3) | 2(1.4) | 0.566 | 0.435(0.025–7.468) |
|  | 2 | 95(61.7) | 112(76.2) | 0.023 | 2.307(1.123–4.740) |
| Noise intensity ≥85 dB(A) | | | | | |
| rs1049216 | CC | 78(67.8) | 83(68.0) |  | 1.0 |
|  | CT+TT | 37(32.2) | 39(32.0) | 0.600 | 0.783(0.314–1.952) |
| rs6948 | AA | 80(69.6) | 84(68.9) |  | 1.0 |
|  | AC+CC | 35(30.4) | 38(31.1) | 0.836 | 0.905(0.352–2.328) |
| Risk genotype | 0 | 34(29.6) | 38(31.1) |  | 1.0 |
|  | 1 | 4(3.5) | 1(0.8) | 0.437 | 0.336(0.021–5.261) |
|  | 2 | 77(67.0) | 83(68.0) | 0.770 | 1.152(0.445–2.987) |

^a^: adjusted for age, sex, education, marriage status, income, working time, noise exposure time, CNE, telephone using time, music listening time, and time go to sleep

Table S2 Stratified analysis by CNE

| SNP | Genotype | Controls, n (%) | Cases, n (%) | P | OR(95%CI)^a^ |
| --- | --- | --- | --- | --- | --- |
| CNE <90 dB(A) | | | | | |
| rs1049216 | CC | 74(60.7) | 89(74.2) |  | 1.0 |
|  | CT+TT | 48(39.3) | 31(25.8) | 0.028 | 0.331(0.124–0.885) |
| rs6948 | AA | 76(62.3) | 91(75.8) |  | 1.0 |
|  | AC+CC | 46(37.7) | 29(24.2) | 0.039 | 0.365(0.140–0.952) |
| Risk genotype | 0 | 46(37.7) | 29(24.2) |  | 1.0 |
|  | 1 | 2(1.6) | 2(1.7) | 0.782 | 0.637(0.026–15.376) |
|  | 2 | 74(60.7) | 89(74.2) | 0.031 | 2.973(1.106–7.997) |
| CNE ≥90 dB(A) | | | | | |
| rs1049216 | CC | 99(67.3) | 106(71.1) |  | 1.0 |
|  | CT+TT | 48(32.7) | 43(28.9) | 0.524 | 0.783(0.369–1.661) |
| rs6948 | AA | 101(68.7) | 107(71.8) |  | 1.0 |
|  | AC+CC | 46(31.3) | 42(28.2) | 0.672 | 0.845(0.387–1.844) |
| Risk genotype | 0 | 45(30.6) | 42(28.2) |  | 1.0 |
|  | 1 | 4(2.7) | 1(0.7) | 0.513 | 0.396(0.025–6.337) |
|  | 2 | 98(66.7) | 106(71.1) | 0.665 | 1.189(0.544–2.598) |

^a^: adjusted for age, sex, education, marriage status, income, working time, noise exposure time, noise intensity exposure, telephone using time, music listening time, and time go to sleep

Table S3 The best combination models identified by MDR

| No. | Best model* | Training balanced  accuracy (%) | Testing balanced  accuracy (%) | P | Cross-validation  consistency |
| --- | --- | --- | --- | --- | --- |
| 1 | 4 | 0.5419 | 0.5088 | 0.3770 | 8/10 |
| 2 | 1, 6 | 0.5683 | 0.5055 | 0.3770 | 6/10 |
| 3 | 2, 5, 6 | 0.6012 | 0.5052 | 0.6230 | 5/10 |
| 4 | 3, 4, 5, 6 | 0.6446 | 0.4898 | 0.8281 | 4/10 |

*1-6 represents rs630003, rs523104, rs13006529, rs1049216, rs1042891, rs3181309
